# Interleukin-6 overexpression and elevated granulocyte-to-lymphocyte ratio indicate hepatic stress in experimental group a Streptococcus sepsis

**DOI:** 10.1007/s00430-025-00826-2

**Published:** 2025-04-03

**Authors:** Valerie Brunsch, Wendy Bergmann-Ewert, Brigitte Müller-Hilke, Johann Aleith

**Affiliations:** Core Facility for Cell Sorting and Cell Analysis, Rostock University Medical Center, Rostock, Germany

**Keywords:** Infection, Sepsis, Group a streptococcus, Cytokines, Mouse model, Flow cytometry

## Abstract

Group A Streptococcus (GAS) is a pathogen that is capable of colonizing various infection sites and can potentially elicit an inadequate immune response that will lead to sepsis. The processes underlying this misdirected immune reaction remain poorly understood, and reliable biomarkers for indicating impending organ failure during sepsis are still missing. The present study aims to identify parameters that can predict the onset of end-organ damage in the course of sepsis. To that extent, we investigated key aspects of the immune response in early-phase sepsis following infection of different tissues in a mouse model, using Brefeldin A to link cytokine production to specific cell types through multi-parameter flow cytometry. Subcutaneous and intravenous GAS infections resulted in clinical sepsis, which was paralleled by peripheral blood lymphopenia. Intravenous infection in particular was associated with a higher bacterial burden in the liver that strongly correlated with an increased granulocyte-to-lymphocyte ratio of the peripheral blood. Strikingly, IL-6 overexpression was more pronounced in intravenous infection and strongly correlated with hepatic stress, indicated by elevated bacterial loads in the liver. Collectively, our data highlight the potential utility of IL-6 in conjunction with an elevated granulocyte-to-lymphocyte ratio as promising early indicators of concomitant liver stress in sepsis.

**Supplementary Information:**

The online version contains supplementary material available at 10.1007/s00430-025-00826-2.

## Introduction

Sepsis is a life-threatening condition caused by a dysregulated response of the body to infection [42]. Organ dysfunction, e.g. liver failure, is a severe complication in the course of sepsis that impacts on the clinical outcome considerably [54]. The liver plays a critical role in the immune response during sepsis through its production of cytokines and acute-phase proteins, bacterial clearance, and metabolic regulation [44]. Acute liver injury caused by sepsis disrupts these functions, leading to hepatitis, cholestasis, and coagulopathies [14]. These changes result from excessive inflammatory cytokine production, also known as a “cytokine storm”, lymphocyte apoptosis, and neutrophil recruitment to the liver [12, 13, 50]. Lymphopenia is further sustained by the suppression of lymphoid progenitor cells in the bone marrow [48]. Approximately 30 million people worldwide are affected by sepsis each year, with one-fifth of them succumbing to the illness [9]. This high mortality rate makes sepsis the leading cause of death in intensive care units and prompted the World Health Organization to classify sepsis as a global health priority [23, 36].

One pathogen capable of inducing sepsis is Group A Streptococcus (GAS). Via different infection sites, it causes a wide spectrum of clinical manifestations from mild entities such as pharyngitis and pyoderma to invasive and potentially life-threatening infections and toxin-mediated diseases. Despite the apparent control facilitated by antibiotic therapies [3], severe diseases such as necrotizing fasciitis, pneumonia, and Streptococcus-induced toxic shock syndrome (STSS) continue to present challenges in modern medicine [6, 37, 41]. Moreover, the incidence of GAS infections has shown an upward trend in industrialized nations since the 1980s, emphasizing the critical need for better understanding the pathophysiological dynamics and revised medical interventions [11, 22, 26, 27]. To this day, the development of a GAS vaccine remains elusive due to gaps in understanding the intricate interplay between virulence factors and host immune responses [51].

Numerous studies have attempted to identify reliable biomarkers for sepsis, though their clinical utility remains limited due to insufficient specificity and sensitivity [2, 34]. Current interest lies in the overexpression of cytokines such as IL-6, IL-10, and CCL2 due to their reported correlation with sepsis severity [30, 46, 49]. To identify cytokines indicating organ failure in sepsis and their cellular source, we here used a mouse model in combination with in vivo application of Brefeldin A. This compound immobilizes cytokines within the cytosol, enabling their detection through intracellular labelling [20, 24]. Cytokine profiles were subsequently analyzed using multi-parametric flow cytometry.

Moreover, using subcutaneous, intravenous, and intranasal infection routes allowed us to investigate whether the immune response in early GAS-induced sepsis is generalized or dependent on the primary site of infection. By establishing a connection between cellular changes and cytokine expression patterns with disease severity and bacterial burden in organs, we ultimately sought to distinguish markers for imminent organ stress during sepsis in the initial phase of disease manifestation, when clinical signs are not pathognomonic.

## Materials and methods

### Animals

C57BL/6J mice were initially obtained from Charles River. They were bred in the animal care facility (Rudolf Zenker Institute for Experimental Surgery, Rostock University Medical Center) under specific germ-free conditions. Aged between 6 and 8 weeks, male animals were transferred to individually ventilated cages and kept on a 12-h light/dark cycle, an ambient temperature of 22 ± 2 °C, and 50 ± 20% humidity for infection experiments. Food and water were provided ad libitum. Animal experiments were reviewed and approved by the ethics committee of the State Department for Agriculture, Food Safety, and Fishery in Mecklenburg-Western Pomerania under the file reference number 7221.3-1-042/22.

### Bacteria

*Streptococcus pyogenes* (Group A Streptococcus, GAS) strain AP1 of the *emm1* (M1) serotype was originally acquired from the World Health Organization Collaborating Center for Reference and Research on Streptococci (Prague, Czech Republic). Bacteria were thawed onto Columbia agar plates containing 5% sheep blood (Becton Dickinson, Franklin Lakes, NJ, USA) and were cultured overnight, followed by storage at 4 °C for up to three weeks. For individual experiments, three colonies were picked from the plate, suspended in Todd-Hewitt broth (THB, Becton Dickinson) and incubated under microaerobic conditions for 16 h at 37 °C and 5% CO_2_. The suspension was then diluted 20-fold in THB followed by incubation until an exponential phase of growth was reached after another 3 h, which was confirmed by measuring the optical density. Afterwards, bacteria were washed three times with PBS (Thermo Fisher, Waltham, MA, USA) and diluted to match an optical density of 0.5, which is equivalent to 10^8^ colony-forming units (CFU) according to our experience. Confirmation of the applied CFU was followed by counting serially diluted suspensions cultivated overnight.

### Pathogen inoculation and quantification

Mice were randomly divided into groups with three different application routes of either PBS (control group, CON) or bacteria (infection group, INF) using a six-sided dice-based random number generator. The application was either performed subcutaneously in the neck, intravenously into the lateral tail vein, or intranasally into both nostrils under 5% isoflurane anaesthesia. As performed in previous studies [39, 52], unpublished in-house data), 10^7^ CFU GAS in 200 µL PBS were used for subcutaneous, 0.5 × 10^6^ CFU in 100 µL PBS for intravenous and 10^8^ CFU in 20 µL PBS for intranasal infection while control animals received only the respective volumes of PBS via the same routes. After 24 h, 10 µL of EDTA-anticoagulated blood was drawn by puncturing the saphenous vein and incubated on agar plates overnight. Colonies of beta-hemolytic bacteria were counted the next day. After euthanasia, homogenized spleen and liver tissue as well as cardiac blood and BAL samples were plated on blood agar plates and incubated overnight.

### Clinical scoring and application of Brefeldin A

Mice were scored at least twice a day for sepsis symptoms for a maximum of 48 h or until humane endpoints were reached as established in previous studies [40, 52]. The evaluation included weight loss, general condition, spontaneous behaviour, and signs of infection. In each category, 0, 5, 10, or 30 points were assigned and summed up at each observation time point to estimate sepsis activity, with 0 points equalling no clinical manifestation, 5 points low grade, 10 to 25 points moderate, and 30 and more points severe disease burden. Humane endpoints were defined to avoid severe disease activity and minimize suffering, including coldness, apathy, necrotizing fasciitis, and open wounds at infection sites. Infected animals received 0.1 mg/mL tramadol (Ratiopharm) in their drinking water for analgesia after bacterial inoculation. Six hours prior to the experimental end point (i.e. 42 h post infection), mice were administered a weight-adapted dose of Brefeldin A (BFA, Sigma, St. Louis, USA) intraperitoneally. Animals received 10 mg per kg body weight of a stock solution containing 20 mg BFA per mL dimethyl sulfoxide, diluted 20-fold in PBS.

### Euthanasia and organ extraction

Forty-eight hours post infection, mice underwent final anaesthesia with an intraperitoneally administered dose of 150 mg ketamine (Pharmanovo) and 10 mg xylazine (Bayer) per kg body weight. Following confirmation of full anaesthesia, mice were exsanguinated by cardiac puncture and then euthanized by cervical dislocation. Blood was collected with EDTA as an anticoagulant. Spleen, liver, and femora were extracted and stored in autoMACS Running Buffer (RB, Miltenyi Biotec, Bergisch Gladbach, Germany), containing 5 µg/mL BFA for the short-term storage of spleen and liver samples. For intranasally infected animals only, a bronchoalveolar lavage (BAL) was performed as previously described [45]. In brief, the trachea was exposed, incised, and punctured with a larynx tube, followed by injection of 0.8 mL RB via the larynx tube and aspiration.

### Single cell analysis by flow cytometry

Erythrocyte lysis was performed on 200 µL anticoagulated cardiac blood using 4 mL of an in-house NH_4_Cl lysis buffer. After ten minutes of incubation with gentle agitation at room temperature, blood cells were centrifuged, and the pellet was suspended in RB. Splenocytes were obtained by passing the spleen through a 70 μm strainer that was subsequently rinsed with RB. After centrifugation of the suspension, the pellet was suspended in 5 ml Red Blood Cell (RBC) Lysis Buffer (BioLegend) and left on ice for 5 min. To stop the lysis, 20 mL RB was added, followed by centrifugation and suspension in RB. For liver processing, 400 mg of cut-up liver was added to 2 mL of a pre-heated solution of 1 mg collagenase/dispase (Sigma) per ml Roswell Park Memorial Institute Medium 1640 (RPMI, Pan-Biotech, Germany) and digested at 37 °C for 30 min on a magnetic stirrer set to 350 rpm. The liver was then processed using the same protocol as for the spleen. For bone marrow extraction, the epiphyses were removed from the femora, and the bones were placed into perforated tubes within an outer tube. After centrifugation at 10,000 g for 15 s (according to Amend et al., [1], the bone marrow was treated using the same lysis protocol as for the spleen and liver.

Cells were counted using a haemocytometer, and 500,000 cells from each suspension were incubated with ZombieNIR (BioLegend) at room temperature for 20 min. Cells were then washed and suspended in 25 µL RB. A blocking mix containing fetal calf serum (FCS, PANbiotech), TruStain FcX (BioLegend), and Monocyte Blocker (BioLegend) was added to prevent nonspecific binding and incubated on ice in the dark for 10 min. Subsequently, an antibody mixture containing (anti-)CD3:BV480 (clone: 17A2), F4/80:APC/R700 (BD, T45-2342), CD8:PerCP/Vio700 (REA601), CD86:APC/Vio 770 (Miltenyi Biotec, PO3.3), CD127:eFluor 450 (Thermo Fisher Scientific, A7R34), B220:BV510 (RA3-6B2), CD80:BV605 (16-10A1), CD4:BV650 (GK1.5), CD11b: BV750 (M1/70), Gr-1:SparkBlue550 (RB6-8C5), CD25:PE/Dazzle594 (3C7), CD11c: PerCP (N418), CD49b: PerCP/Cy5.5 (HMα2), CD45:APC/Fire810 (30-F11), and I-A/I-E: PE/Fire640 (BioLegend, M5/114.15.2) was added to blood, liver, spleen, and BAL samples and incubated on ice in the dark for 20 min. The antibody mixture for BAL samples also contained IgM: BV711 (BioLegend, RMM-1). For bone marrow, an antibody mixture of CD3:BV570 (17A2), CD105:BV786 (MJ7/18), CD44:APC/R700 (BD, IM7), CD38:VioBlue (Miltenyi Biotec, REA616), c-Kit: BV421 (QA17A09), B220:BV510, CD90.2:BV605 (30-H12), CD11b: BV750, Gr-1:SparkBlue 550, Flk2:PE (A2F10), Sca-1:PE/Dazzle594 (D7), CD127:PE/Cy5 (A7R34), CD106:PerCP/Cy5.5 (429), SLAM: PE/Cy7 (TC15-12F12.2), CD73:APC (TY/11.8), CD34:AlexaFluor647 (SA376A4), CD48:APC/Fire750 (HM48-1), and I-A/I-E: PE/Fire640 (BioLegend) was added and incubated as described above. Cells were washed, and bone marrow cells were suspended in 200 µL RB. For intracellular staining, blood, liver, and spleen samples were suspended in 0.5 mL fixation buffer (BioLegend) and incubated in the dark at room temperature for 20 min. After centrifugation, cells were washed three times with 0.5 mL intracellular staining permeabilization wash buffer (BioLegend). The resuspended cells were incubated with blocking mix at room temperature for 10 min. Then, an intracellular staining antibody mixture containing TNFα:BV421 (MP6-XT22), IL-17 A: BV785 (TC11-18H10.1), CCL2:PE (2H5), IL-10:PE/Cy7 (JES5-16E3), IFNγ:AlexaFluor647 (BioLegend, XMG1.2), IL-6:FITC (REA1034), and CCL3:APC (Miltenyi Biotec, REA355) was added and incubated at room temperature for 20 min. Cells were washed with 2 mL perm buffer and suspended in 200 µL RB.

Data acquisition was performed on a Cytek^®^Aurora spectral flow cytometer running on the SpectroFlo software v3.3 (Cytek Biosciences, Fremont, CA, USA). Data analysis was conducted using the FlowJo software v10.7.1.

### Statistical analysis

Data analysis and visualization were performed using GraphPad Prism 8.0.2 and SRplot [47]. Data sets were tested for Gaussian distribution using the Shapiro-Wilk test. Comparisons between two groups were made using an unpaired two-sided t-test for parametric data and the Mann-Whitney test for non-parametric data. For tests involving multiple comparisons, the Kruskal-Wallis test with Dunn’s correction or two-way ANOVA with post hoc Tukey’s test were conducted. Correlation analysis was performed via Spearman correlation. A p-value of < 0.05 was considered statistically significant.

## Results

### Clinical deterioration of GAS-induced sepsis was influenced by the route of infection

**Fig. 1:**
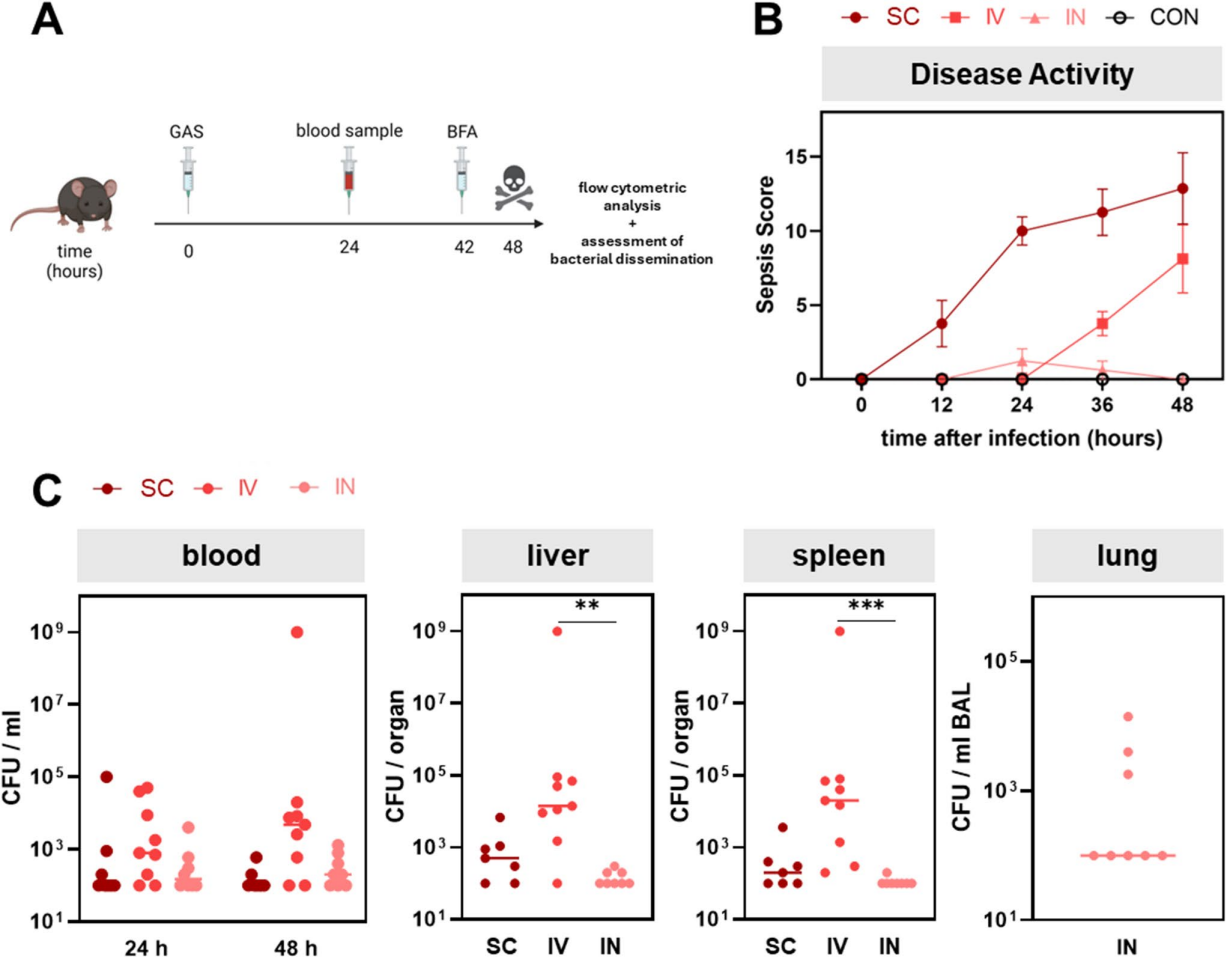
Clinical disease burden and bacterial dissemination in Group A Streptococcus (GAS)-infected mice. (**A**) Experimental design depicting the infection protocol of C57BL/6J mice with GAS via subcutaneous (SC, *n* = 8), intravenous (IV, *n* = 9), and intranasal (IN, *n* = 8) routes with respective control groups receiving PBS. (**B**) Disease activity scores over 48 h post infection for SC, IV, and IN routes. Dots and error bars display mean sepsis scores and standard error of the mean, respectively. (**C**) Bacterial dissemination in blood and organs quantified by colony-forming units (CFU) per ml or per organ. Dots indicate individual counts of CFU per mouse. Bars represent median values. Groups were compared using the Kruskal-Wallis test with Dunn’s multiple comparisons test (**p < 0.01, ***p < 0.001)

To determine whether different infection routes elicit distinct immune responses, C57BL/6J mice were infected with GAS via subcutaneous, intravenous, or intranasal routes. Corresponding control animals received PBS. Peripheral blood samples from all groups were taken at 24 and 48 h to assess bacterial load. At 42 h (6 h prior to experimental end point), mice were intraperitoneally administered a weight-adapted dose of BFA. Animals were then euthanized, and their organs were harvested for subsequent analyses (Fig. 1A).

In order to investigate whether the different infection routes resulted in varying clinical progressions, we scored sepsis severity via clinical manifestations, such as weight loss and abnormal behaviour or appearance. Indeed, when comparing sepsis scores between infection groups and over time, significant differences were observed (*p* < 0.001, two-way ANOVA, Fig. 1B). In detail, subcutaneously infected animals exhibited immediate weight loss and clinical signs of infection, plateauing at 24 h. In contrast, intravenously infected mice showed a delayed progression without any clinical manifestations for the first 24 h, with a steep increase in disease activity thereafter. At the final observation point at 48 h, disease activity was comparable between the subcutaneous and intravenous routes (*p* = 0.179, two-sided t-test). Surprisingly, intranasally infected mice did not display significant signs of infection at any time.

Bacterial dissemination was assessed in the peripheral blood at 24 and 48 h and in homogenized organs and BAL fluids at endpoint. Despite the differences in clinical progression, bacterial load in the blood was similar across all infection routes at both time points (Fig. 1C). As for peripheral organs, intravenously infected mice exhibited the highest bacterial dissemination, while intranasally infected animals had only minimal bacterial colonization.

In summary, subcutaneous and intravenous infections culminated in comparable sepsis manifestation at the experimental endpoint, even though differences occurred in kinetics and bacterial disseminations. In contrast, intranasal infection did not elicit sufficient disease progression to effectively model sepsis. These findings indicate that while bacteraemia was present across all infection routes, the primary site of GAS infection impacted on clinical progression.

### Increased granulocyte-to-lymphocyte ratio in the blood of septic animals correlated with high bacterial burden in the liver

**Fig. 2:**
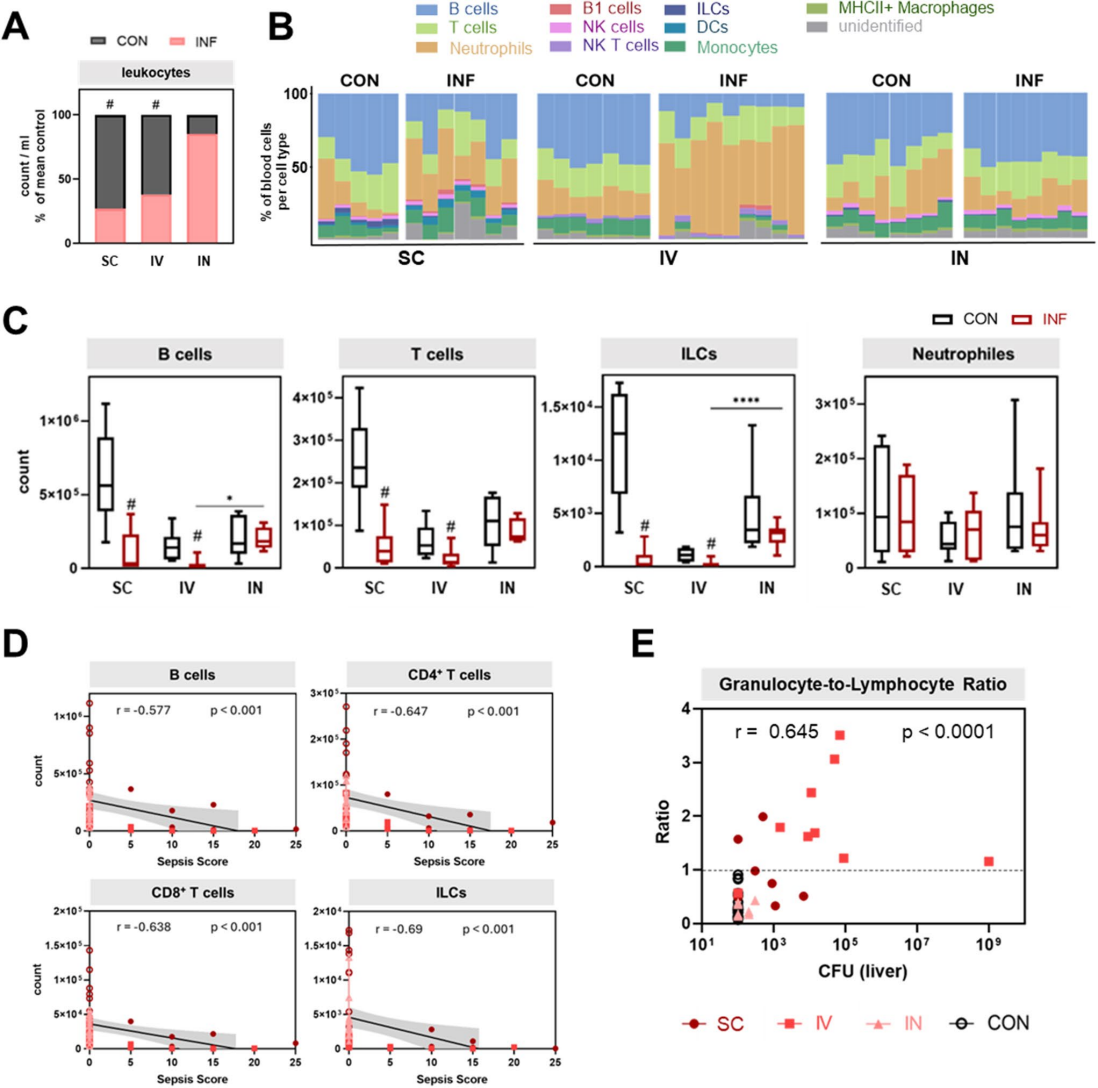
Immune cell alterations in the blood after GAS infection. (**A**) Absolute leukocyte counts of infected animals (INF) as a fraction of counts in control animals (CON). Interleaved bars indicate mean values. (**B**) Stacked bar plots depict immune cell compositions. Columns represent individual samples. (**C**) Absolute counts of B cell, T cell, ILC, and neutrophil populations are shown. Data of control and infected animals are presented for each infection route. Box plots show median values and interquartile range. Mann-Whitney test was used for comparison between control and infected animals (#*p* < 0.05). Kruskal-Wallis test with Dunn’s multiple comparisons test was applied for comparisons between infection routes (*p < 0.05, ****p < 0.0001). (**D**) Correlation analysis of leukocyte population counts with sepsis score is presented as a linear regression graph with a 0.95 confidence interval. Spearman correlation coefficient (r) is indicated. Dots represent individual animals. (**E**) Granulocyte-to-lymphocyte ratio of the peripheral blood was correlated with bacterial load in the liver using Spearman correlation

After establishing that different infection routes caused distinct clinical progressions, we investigated the underlying immune responses by assessing the respective peripheral blood immune cell landscapes. GAS infection in general led to reduced leukocyte counts across all groups, with significant leukopenia observed in subcutaneous and intravenous, but not intranasal infections (Fig. 2A).

To identify cell types affected by leukopenia, we next analyzed immune cell compositions using flow cytometry, applying the gating strategy for blood samples shown in Supplementary Fig. 1A. Subcutaneously and intravenously infected mice showed a relative increase of the neutrophil population at the expense of the B cell fraction (Fig. 2B). Absolute counts confirmed reductions in B cells, T cells, and innate lymphoid cells (ILCs, Fig. 2C), suggesting that lymphopenia was the main cause of leukocyte composition alterations. Although there was also a decrease in macrophage counts, the alterations in this low-frequency population affected the peripheral immune landscape only marginally (Supplementary Fig. 1B). Conversely, neutrophil counts remained stable during this phase of infection, as well as natural killer (NK) cells, natural killer T cells (NKTs), dendritic cells (DCs) and CD11b^+^B220^+^ B1 cells. Correlation analyses between leukocyte population counts and sepsis scores revealed a negative correlation of B cell, T cell, and ILC numbers with sepsis severity (Fig. 2D), underscoring the strong interrelationship between lymphocyte depletion and disease progression. Finally, we asked whether the granulocyte-to-lymphocyte ratio could be linked to the bacterial load in the liver as an indicator of organ stress and indeed, an elevated ratio (> 1) correlated significantly with a higher bacterial burden (Fig. 2E). These increased ratios were almost exclusively observed after intravenous infection, hinting at a link between reduced circulating lymphocytes and peripheral bacterial dissemination.

In summary, the early phase of clinically apparent sepsis was characterized by peripheral blood lymphoid cell depletion, resulting in an elevated granulocyte-to-lymphocyte ratio, which in turn showed a significant association with both disease severity and bacterial burden in the liver.

### Intravenous infection resulted in increased granulocyte infiltration and B lymphopenia in the liver

**Fig. 3:**
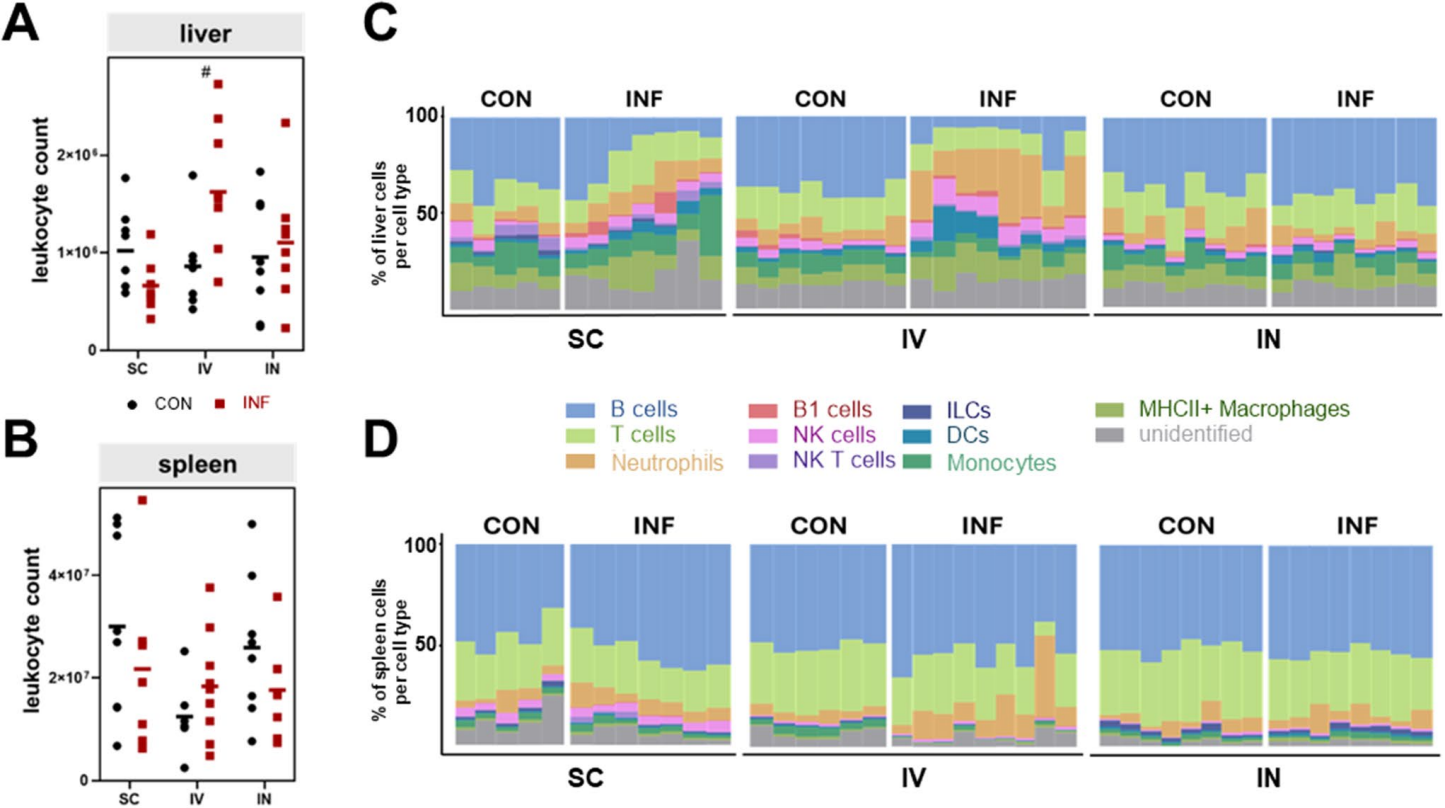
Immune cell alterations in peripheral organs after GAS infection. Absolute leukocyte counts in liver (**A**) and spleen (**B**). Data of control and infected animals are presented for each infection route. Dot plots represent individual samples and lines depict mean values. Mann-Whitney test was used for comparisons between control and infected animals (#*p* < 0.05). Immune cell compositions of the liver (**C**) and spleen (**D**) are depicted in stacked bar plots for various cell types. Columns represent individual samples

Next, we explored the effects of GAS infection on the immune cell landscapes in the liver, spleen and lung (i.e. bronchoalveolar lavage, for intranasal infection only). For spleen and liver, we implemented the gating strategy outlined in Supplementary Fig. 2A to assess both absolute immune cell numbers as well as their relative distribution. Notably, intravenous infection resulted in a significant increase in hepatic leukocyte infiltration, while the spleen remained unaffected (Fig. 3A and B).

In intranasally infected animals, using the gating strategy shown in Supplementary Fig. 3A, bronchoalveolar lavage revealed significant leukocyte migration into the lower respiratory tract despite the lack of clinical symptoms, confirming an adequate immune response following mucosal infection. In detail, significant increases in relative cell numbers were noted for B cells, T cells, ILCs, and NK cells, while the fraction of neutrophils was reduced (Supplementary Fig. 3B).

In the livers of subcutaneously and intravenously infected mice, we observed immune cell alterations similar to those in the blood. Especially in intravenous infection, there was a relative decrease in B cells and an increase in neutrophils (Fig. 3C). Again, the percentages of B cells correlated negatively with the respective sepsis score (Supplementary Fig. 3B and C). In contrast, the fraction of DCs increased after infection and positively correlated with disease activity. In the spleens however, the immune cell compositions remained largely stable post infection (Fig. 3D), with significant alterations restricted to low-frequency populations. In detail, ILCs were significantly depleted, while CD11b^+^B220^+^ B1 cell fractions increased in subcutaneous and intravenous infection (Supplementary Fig. 3E). Both alterations correlated strongly with the sepsis score (Supplementary Fig. 3F).

These findings suggest that during the early phase of sepsis, splenic immune cell proportions remain largely unaffected by both subcutaneous and intravenous GAS infections. In contrast, the liver exhibits distinct immune alterations, with intravenous infection leading to pronounced hepatic leukocyte infiltration.

### Early sepsis induced depletion of common myeloid and lymphoid progenitor cells in the bone marrow

**Fig. 4:**
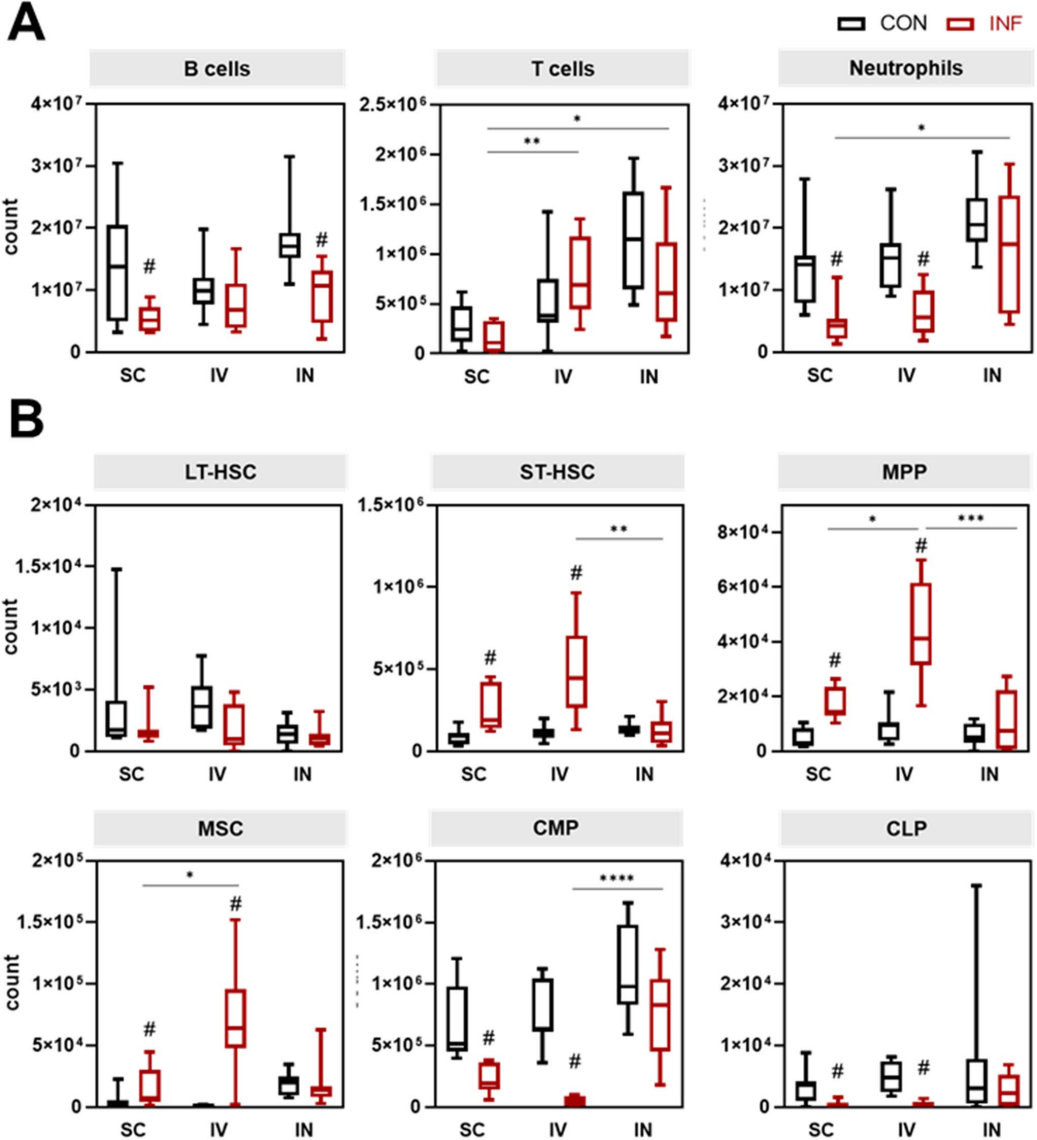
Changes in leukocytes (**A**) and their progenitor cells (**B**) in the bone marrow after GAS infection. Data of control and infected animals are presented for each infection route. Box plots show median values and interquartile range. Mann-Whitney test was used for comparison between control and infected animals (#*p* < 0.05). Kruskal-Wallis test with Dunn’s multiple comparisons test was applied for comparisons between infection routes (*p < 0.05, **p < 0.01, ***p < 0.001, ****p < 0.0001)

In order to link the peripheral immune response to the stem and progenitor cell landscape, we analyzed the bone marrow compartment of infected mice using the gating strategy shown in Supplementary Fig. 4A. To that end, we examined the main lineage leukocyte populations in the bone marrow in absolute (Fig. 4A) and relative cell counts (Supplementary Fig. 4B). Interestingly, B cell reduction was observed across all infection routes, yet reached significance only following subcutaneous and intranasal infection. Similarly, neutrophil depletion occurred and was paralleled by a negative correlation of sepsis activity with Gr-1 expression on neutrophils (Supplementary Fig. 4C). Since Gr-1 serves as a marker of neutrophil maturity [8], we further analyzed the population of immature Gr-1^−^ neutrophils, applying the gating strategy shown in Supplementary Fig. 5A. Compared to mature polymorphonuclear Gr-1^+^ neutrophils, immature neutrophils were significantly enriched in the blood of septic animals, while their increase in the spleen and liver was less pronounced (Supplementary Fig. 5B). Cytokine analysis further indicated reduced functionality, as immature neutrophils exhibited a universal decrease in IL-6 expression and partial decline in TNFα and IFNγ levels in the bloodstream and liver (Supplementary Fig. 5C).

Next, we investigated alterations of the stem and progenitor cells. Numbers of long-term hematopoietic stem cells (LT-HSCs) were unaffected by infection, but short-term hematopoietic stem cells (ST-HSCs), mesenchymal stem/stromal cells (MSCs), and multipotent progenitor cells (MPPs) increased significantly following subcutaneous and intravenous infections (Fig. 4B). These effects were more pronounced in intravenously infected mice compared to the other infection routes. Conversely, common myeloid progenitors (CMPs) and common lymphoid progenitors (CLPs) significantly decreased after both subcutaneous and intravenous infection.

To link alterations in the bone marrow compartment to shifts in the peripheral immune cell landscape, we performed correlation analyses. Supplementary Fig. 6 illustrates that the sepsis-induced accumulation of bone marrow MPPs and ST-HSCs significantly correlated with a decrease in CMPs, CLPs, and bone marrow neutrophils and was also associated with peripheral lymphocyte depletion. Furthermore, an increase in peripheral immature neutrophils was significantly associated with this disruption in hematopoiesis.

Our data suggest that ongoing emergency hematopoiesis was characterized by an exhausted reservoir of progenitor cells from both myeloid and lymphoid lineages, which coincided with a failure to replenish depleted peripheral lymphocytes while leading to the release of immature neutrophils with reduced effector functions.

### B cells were key drivers of cytokine production and IL-6 overexpression in peripheral blood indicated hepatic stress in sepsis

**Fig. 5:**
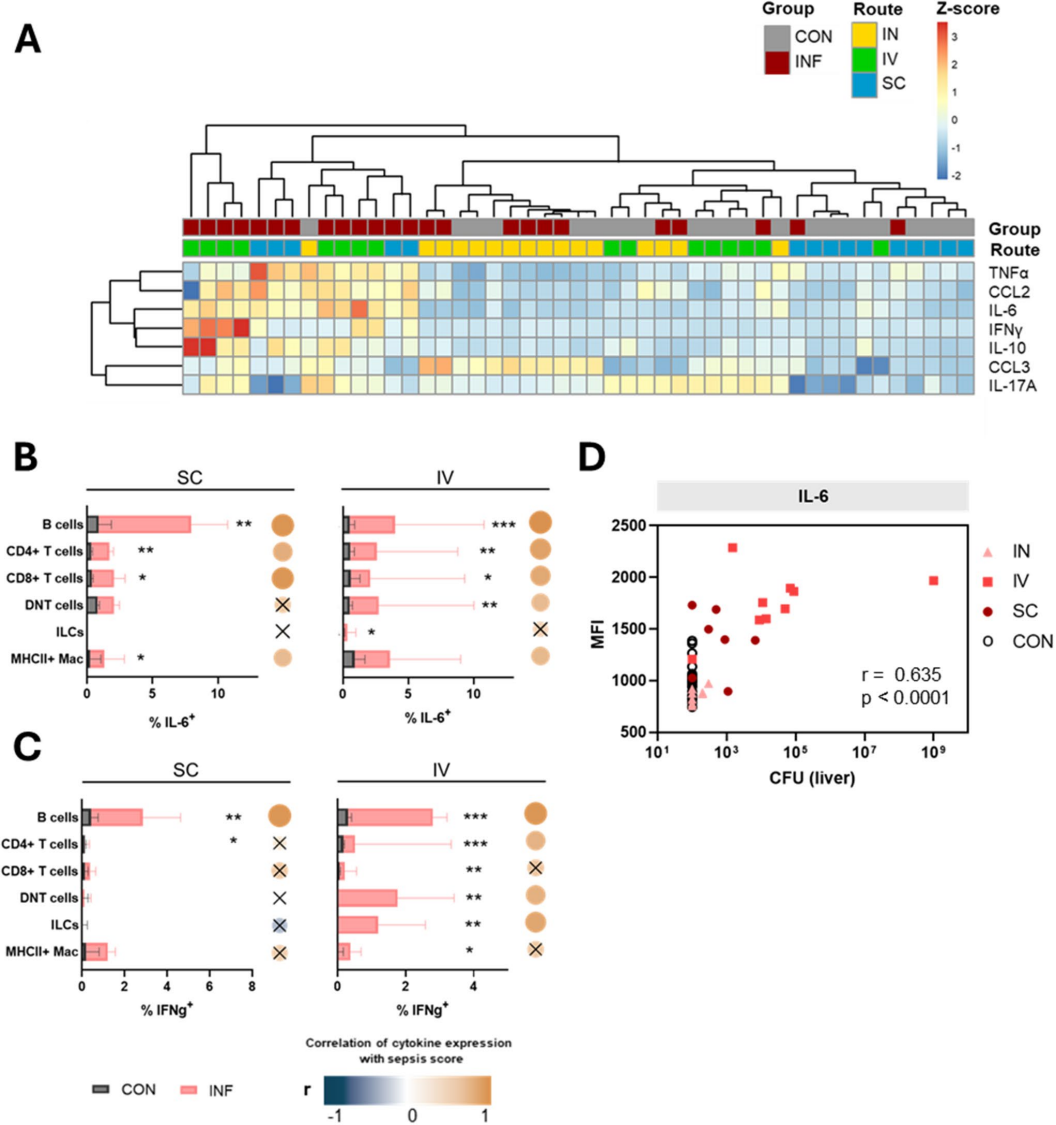
Cytokine expressions in the blood after GAS infection. (**A**) Heat map of color-coded z-scored mean fluorescence intensity of cytokines in live leukocytes. Percentage of IL-6^+^ (**B**) and IFNγ^+^ (**C**) cells among immune cell populations are demonstrated for subcutaneous and intravenous infection. Data of control and infected animals are presented for each infection route. Superimposed bar plots show median values with interquartile range. Mann-Whitney test was used for comparison between control and infected animals (*p < 0.05, **p < 0.01, ***p < 0.001). Dots represent Spearman correlation analysis of cytokine expression with sepsis score, with correlation coefficient (r) indicated by color and *p* > 0.05 marked x. (**D**) Bulk IL-6 expression was correlated with bacterial load in the liver using Spearman correlation

Next, we examined the cellular origins of cytokines during early sepsis. To this end, we generated broad cytokine profiles at a single cell level and concentrated on a selection of pro- and anti-inflammatory cytokines as well as chemokines. Analyzing general cytokine expressions by all peripheral blood leukocytes, we found only a non-specific upregulation of CCL3 in both intranasally infected and their respective control mice (Fig. 5A and Supplementary Fig. 7A). Due to this overall inconspicuous cytokine profile, we thus focused on subcutaneous and intravenous infections. Here, we observed elevated expressions of IL-6, IFNγ, CCL2, TNFα, and IL-10 in blood leukocytes. Notably, IFNγ-expressing cells were more prevalent in intravenous infection.

In order to filter for clinically relevant cytokine dynamics, we performed correlation analyses between significant changes in cell type-specific cytokine expressions and the infection route-specific sepsis scores. This way, we identified B cells, T cells, ILCs, and MHCII^+^ macrophages as key contributors to cytokine production (Supplementary Table 1). No relevant changes were observed for IL-10, IL-17A, and CCL3 in the cell type-specific analyses. However, IL-6^+^ cells were significantly enriched in infected animals across nearly all cell types and regardless of infection route, and their frequency correlated positively with the sepsis score (Fig. 5B). Of note, the highest proportion of IL-6 expressing cells was found in the B cell population. IFNγ expression in subcutaneous infection was significantly increased in B cells only, while intravenously infected animals demonstrated elevated IFNγ levels in B cells, T cells, ILCs, and macrophages (Fig. 5C). Consequently, intravenous infection was characterized by a strong correlation between IFNγ expression and the sepsis score for the mentioned cell types. CCL2 levels were enriched in B cells of both subcutaneously and intravenously infected animals, while upregulation of CCL2 in ILCs and macrophages was significant only following subcutaneous infection (Supplementary Fig. 7B). Finally, TNFα overexpression was restricted to B cells and ILCs following subcutaneous infection, while intravenously infected animals remained inconspicuous (Supplementary Fig. 7C).

Next, we investigated how cytokine analysis could aid in predicting organ complications in sepsis. Given the strong correlation between disease activity and IL-6 expression, we further explored the relationship between bulk IL-6 levels and organ stress. Therefore, IL-6 expression was correlated with hepatic bacterial load. We found that intravenously infected animals, which were characterized by increased bacterial accumulation in the liver, exhibited the highest IL-6 levels (Fig. 5D).

In summary, especially peripheral blood B cells produced multiple pro-inflammatory cytokines following both subcutaneous and intravenous infection. IL-6 levels were consistently elevated across various blood lymphocyte populations. Notably, following intravenous infection, excessive overexpression of IL-6 was strongly associated with hepatic stress.

### Intravenous infection led to liver inflammation with an unbalanced expression of IFNγ and IL-10

**Fig. 6:**
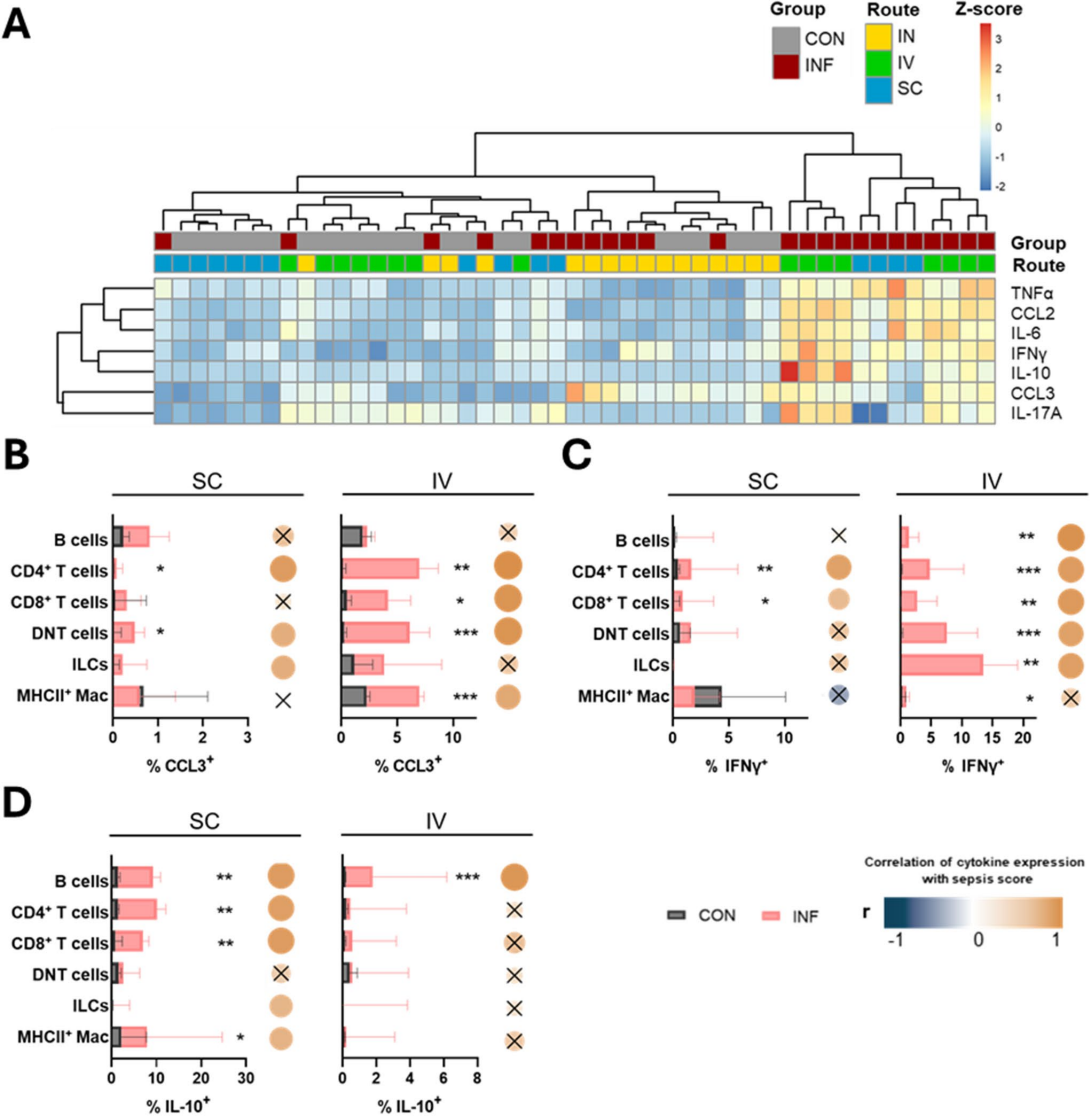
Cytokine expressions in the liver after GAS infection. (**A**) Heat map of color-coded z-scored mean fluorescence intensity of cytokines in hepatic leukocytes. Percentage of CCL3^+^ (**B**), IFNγ^+^ (**C**), and IL-10^+^ (**D**) cells are demonstrated for subcutaneous and intravenous infection. Data of control and infected animals are presented for each infection route. Superimposed bar plots show median values with interquartile range. Mann-Whitney test was used for comparison between control and infected animals (*p < 0.05, **p < 0.01, ***p < 0.001). Dots represent Spearman correlation analysis of cytokine expression with sepsis score, with correlation coefficient (r) indicated by color and *p* > 0.05 marked x

Cytokine expressions among leukocytes were also investigated in peripheral organs. In intranasally infected animals, leukocyte populations from BAL samples during intranasal infections showed elevated levels of CCL3 among T cells and macrophages (Supplementary Fig. 8A). Moreover, B cells and CD4^+^ T cells demonstrated a slight but significant overexpression of IFNγ. Given that there was no discernible cytokine reaction in the lung beyond this (Fig. 6A), these findings suggest a localized mucosal immune reaction in the lower respiratory tract following intranasal infection. In the livers of subcutaneously and intravenously infected animals, bulk MFI levels of CCL3, IFNγ, IL-10, IL-6, CCL2, and TNFα were significantly increased (Fig. 6A, Supplementary Fig. 8B). Interestingly, the tendency towards IL-17A overexpression among liver leukocytes was restricted to intravenously infected mice (Fig. 6A). In contrast, cytokine levels in spleen cells remained unchanged, except for an increase in IFNγ following intravenous infection (Supplementary Fig. 8C). We thus focused further analyses on the liver.

Correlation analyses with the sepsis score again identified lymphocytes and macrophages as key contributors to cytokine overproduction (Supplementary Table 2). When investigating differences between the infection routes, we found an enrichment of CCL3^+^ T cells and MHCII^+^ macrophages following intravenous infection (Fig. 6B). Due to their strong correlation with the sepsis score, this indicates increased chemotactic functions during intravenous infection. Intravenous infection also resulted in significantly elevated levels of IFNγ-expressing cells across all lymphocyte populations (Fig. 6C), which again correlated strongly with the sepsis score. Although there was a minor increase in median IFNγ expression by T cell populations following subcutaneous infections, this did not exceed 2%, rendering it less relevant. Strikingly, IL-10 was only marginally expressed by B cells in intravenously infected mice (Fig. 6D). In contrast, subcutaneous infection led to increased IL-10^+^ B cells, CD4^+^, CD8^+^ T cells, and MHCII^+^ macrophages, all correlating with the sepsis score. This suggests a diminished anti-inflammatory response after intravenous infection.

Collectively, our analysis reveals a strong chemotactic response and a higher pro-inflammatory cytokine burden in the liver following intravenous infection. Increased expression of IFNγ in hepatic lymphocytes was paralleled by insufficient IL-10-mediated anti-inflammatory regulation. This suggests a distinct cytokine imbalance and highlights liver involvement in excessive inflammation.

## Discussion

This study presents the first comparative characterization of immune processes following GAS infection at different sites and contributes to the identification of early-phase sepsis biomarkers with diagnostic value for organ stress. Our findings highlight the impact of the primary infection site on the clinical course of sepsis. While intranasal infection did not result in systemic disease, subcutaneous and intravenous infection caused clinical deterioration at different kinetics yet culminated within 48 h in comparable disease burdens. Given the rapid decline in general health status in intravenously infected mice, our data align with clinical studies linking vascular-origin sepsis to poor outcomes [38, 43]. The mild course of intranasal infection in our model suggests a localized mucosal immune response, making this infection route suitable for representing an immune layout eliciting host resilience.

In both subcutaneous and intravenous infections, we identified critical immune parameters tied to sepsis development. First, we noticed lymphopenia in the blood, which serves as an indicator of early sepsis [5, 13]. Remarkably, B cell counts showed the most significant decrease among leukocyte populations in our experiment. This aligns with a clinical meta-analysis linking reduced B cell numbers to lower survival in sepsis patients [21]. However, our analysis of early-phase sepsis revealed stable neutrophil counts in peripheral blood. Concomitantly, we detected an increased granulocyte-to-lymphocyte ratio, as suggested by Loof et al. [25]. Beyond its known function as a sepsis biomarker, our findings further indicate a strong correlation with the extent of bacterial colonization in the liver. The highest hepatic bacterial burden and most pronounced increase in the granulocyte-to-lymphocyte ratio occurred in intravenously infected animals, suggesting liver stress particularly for this route. Therefore, the granulocyte-to-lymphocyte ratio has the potential to indicate impending organ failure, thus addressing the existing diagnostic gap for sepsis-associated liver damage described by Woznica et al. [54].

Examining leukocyte maturation processes in the bone marrow, we found an enrichment of ST-HSCs and MPPs as similarly described by Morales-Mantilla et al. [33]. However, this upregulation of undifferentiated cells appears insufficient to counteract the peripheral leukocyte deficit, as CLP and CMP counts were reduced in our analysis and correlated with peripheral lymphocyte depletion. Conclusively, while the first line of haematopoiesis remained intact during sepsis, our data suggest an exhaustion of immediate progenitor cells in the bone marrow. Furthermore, we showed a reduction of bone marrow neutrophil counts, with reduced Gr-1 expression as a marker of their maturity [8], indicating emergency granulopoiesis and the deployment of immature neutrophils to peripheral sites [28]. We observed that the immature neutrophil subpopulation exhibited significantly lower expressions of pro-inflammatory cytokines. This impaired functionality may contribute to the dysregulated immune response in sepsis. Emergency recruitment of immature immune cells usually includes monocytes, which transiently express Gr-1 in the bone marrow. The Gr-1 antibody clone used in our study has low affinity for the monocyte marker Ly6C [10], potentially obscuring peripheral neutrophil counts. However, the near-universal IFNγ expression in Gr-1^+^ neutrophils allowed clear distinction from monocytes, which typically lack IFNγ expression. Myeloid-derived suppressor cells were not analyzed, as they cannot be reliably delineated from neutrophils and monocytes using flow cytometry surface markers alone [4]. Together, our analyses of the immune and stem cell landscape suggest an imbalanced immune response skewed toward dysfunctional innate immunity at the expense of adaptive immunity, regardless of infection route.

Our study successfully implemented in vivo Brefeldin A application as a means to identify relevant cytokine-producing cell types in the early phase of sepsis in situ. We observed a strong pro-inflammatory cytokine response in the blood, dominated by IL-6, IFNγ, TNFα, and CCL2, with lymphocytes, particularly B cells, as the primary contributors. Although innate immune cells are typically considered crucial during the early sepsis, our data suggest that cytokine overexpression in sepsis is primarily driven by cells of adaptive immunity. Consistently, B cells are reported to play a pivotal role in the regulation of both innate and adaptive immune responses beyond their function of antibody production [31, 32, 35]. Their indispensability for the initiation of many immune processes, such as pathogen clearance mediated by innates, has been reported before [15]. This holds true even in the context of lymphopenia, where selective apoptosis predominantly affects immature B cells [56], while the surviving mature B cells exhibit a highly inflammatory phenotype [16]. In this setting, a compensatory increase in IL-6 may serve to amplify the inflammatory response despite lymphocyte depletion. Our findings suggest that the innate-like functions of lymphocytes may still be underappreciated and warrant further investigation.

IL-6 is widely recognized as a promising biomarker for sepsis due to its strong correlation with sepsis severity [30, 53]. Our finding that IL-6 is elevated irrespective of the infection focus in clinically apparent sepsis consolidates this knowledge. Especially vascular-origin sepsis is reportedly linked to high mortality and elevated IL-6 levels [38, 43, 55]. In our intravenous infection model, we observed a rapidly progressing, impending lethal outcome driven by hepatic stress. Sepsis-associated acute liver injury was evidenced by a higher bacterial burden, excessive leukocyte chemotaxis, increased emergency granulopoiesis with recruitment of neutrophils to the liver, and heightened pro-inflammatory cytokine expression. While organ involvement is nearly inevitable in sepsis [42], its early detection remains a challenge. Bilirubin, though commonly used to diagnose hepatic failure [7], typically rises only in later stages of disease progression [29]. As liver failure is a critical determinant of sepsis progression [18], our findings suggest that IL-6 overexpression may serve as an early indicator of hepatic stress in sepsis. Thus, utilizing IL-6 as an additional biomarker next to the granulocyte-to-lymphocyte ratio may enable more precise characterization of sepsis subentities, facilitating earlier diagnosis and tailored therapeutic interventions to prevent irreversible organ damage.

Despite our profound insights into the immune response during sepsis originating from different infection sites, this study has some limitations. Our findings are based on a mouse model where sepsis onset was predictable. While this allowed early sampling – before the diagnosis would be made in a clinical setting – future studies should validate these findings using human samples to enhance their translational relevance. Although GAS is a strictly human pathogen, we used a mouse model in an attempt to mirror immune processes. However, the application of a highly pathogenic GAS strain resulted in disease manifestations similar to human infections when inoculating various tissues. Only intranasal application of GAS was not suitable for inducing pneumonia-associated sepsis. Nonetheless, local inflammatory responses in the lung, i.e. leukocyte infiltration and CCL3 overexpression, reflect a successful infection and an effective administration of BFA even in a more limited immune reaction. Additionally, our monomicrobial infection model does not fully replicate the polymicrobial nature that is common in sepsis [17], warranting further studies using sepsis models as described by Korneev [19]. Key cytokines involved in sepsis, such as IL-1β, were excluded from our analysis, as its bioactive form cannot be detected through intracellular staining. Future studies should therefore validate and supplement our findings using methods like ELISA. Additionally, our data represent a single time point at 48 h and should be expanded by cytokine dynamics across different stages of sepsis. Finally, our study’s correlative design limits mechanistic insights, which should be explored in future research.

In conclusion, our study emphasizes the relationship between lymphocyte-driven cytokine expression and sepsis activity regarding organ involvement across different infection routes. Importantly, both IL-6 expression and an increased granulocyte-to-lymphocyte ratio in the peripheral blood correlate strongly with bacterial load in the liver, suggesting that the combination of these two markers may be a robust tool to predict hepatic stress.

## Electronic supplementary material

Below is the link to the electronic supplementary material.

Supplementary Material 1

## Data Availability

The raw data supporting the conclusions of this article will be made available by the authors upon reasonable request.
