# Supplementary material for: Interleukin-6 overexpression and elevated granulocyte-to-lymphocyte ratio indicate hepatic stress in experimental group a Streptococcus sepsis
Source: Med Microbiol Immunol. 2025 Apr 3;214(1):17. doi: 10.1007/s00430-025-00826-2 (PMC11968515; doi:10.1007/s00430-025-00826-2)
Supplement: Supplementary file 1 — Supplementary Material 1 [file 430_2025_826_MOESM1_ESM.docx]

**Interleukin-6 Overexpression and Elevated Granulocyte-to-Lymphocyte Ratio Indicate Hepatic Stress in Experimental Group A Streptococcus Sepsis**

Valerie Brunsch *et al.*

Supporting Information

Contents:

*Supplementary Figures*

**Supplementary Figure 1:** Immune cell landscape in the blood after GAS infection.

**Supplementary Figure 2:** Immune cell landscape in liver and spleen after GAS infection

**Supplementary Figure 3:** Immune cell landscape in the lung after GAS infection.

**Supplementary Figure 4:** Cell landscape in the bone marrow after GAS infection.

**Supplementary Figure 5:** Alterations in neutrophil populations after GAS infection.

**Supplementary Figure 6:** Correlation analysis of the bone marrow compartment and the peripheral immune landscape.

**Supplementary Figure 7:** MFI of cytokines in the blood after GAS infection.

**Supplementary Figure 8:** Cytokine analysis in peripheral organs.

*Supplementary Tables*

**Supplementary Table 1:** Correlation analysis of cytokine expression by leukocyte populations in the blood with sepsis score in subcutaneous (SC) and intravenous (IV) infection.

**Supplementary Table 2:** Correlation analysis of cytokine expression by leukocyte populations in the liver with sepsis score in subcutaneous and intravenous infection.


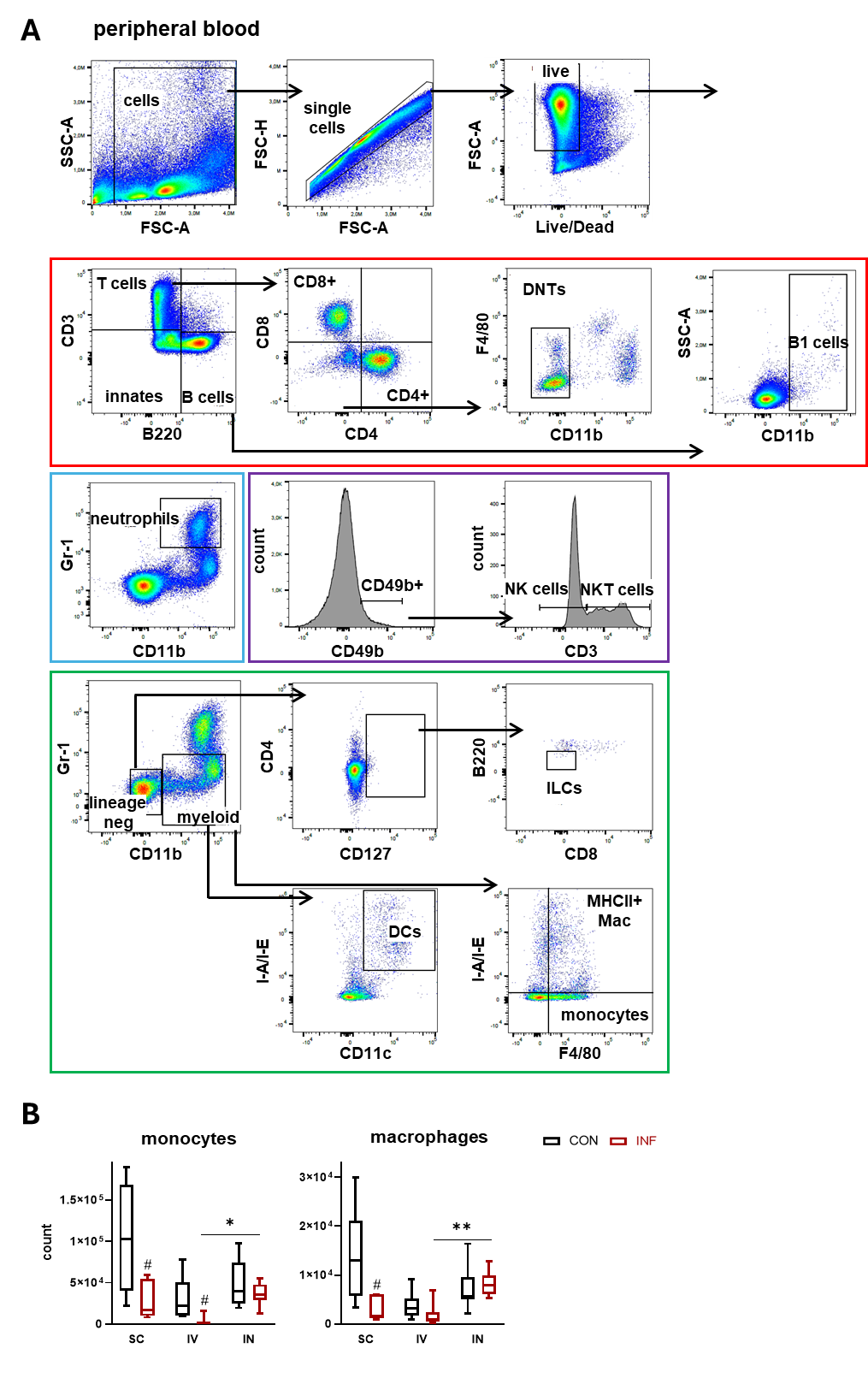


**Supplementary Figure 1. Immune cell landscape in the blood after GAS infection. (A)** Gating strategy for flow cytometry of the peripheral blood. **(B)** Absolute counts of macrophages. Data of control and infected animals are presented for each infection route. Box plots show median values and interquartile range. Mann-Whitney test was used for comparisons between control and infected animals (#p < 0.05). Kruskal-Wallis test with Dunn’s multiple comparisons test was applied for comparisons between infection routes (*p < 0.05, **p < 0.01).


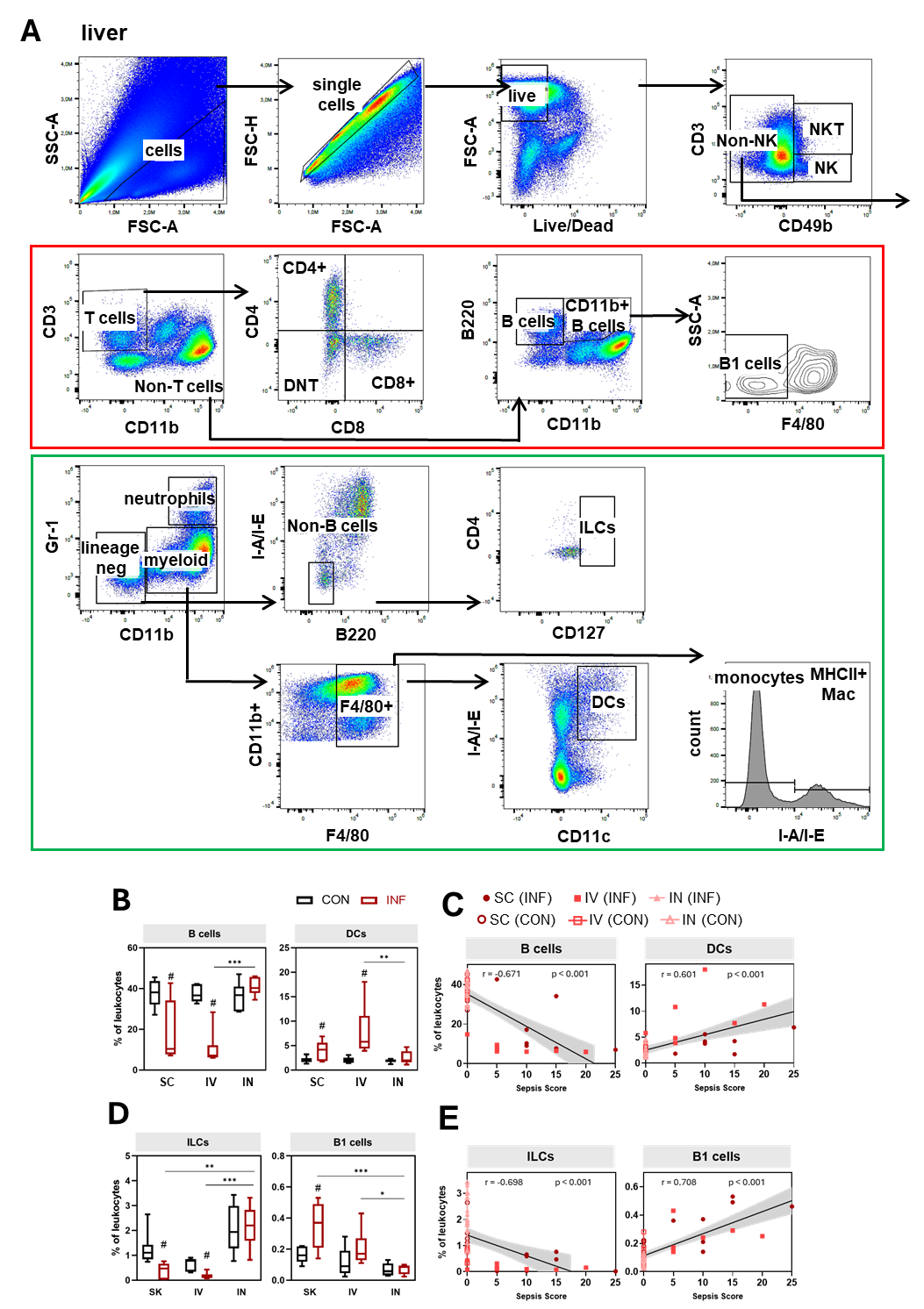


**Supplementary Figure 2. Immune cell landscape in liver and spleen after GAS infection.** **(A)** Gating strategy for flow cytometry of the liver. Fractions of leukocyte cell populations in liver **(B)** and spleen **(D)** are shown. Data of control and infected animals are presented for each infection route. Box plots show median values and interquartile range. Mann-Whitney test was used for comparison between control and infected animals (#p < 0.05). Kruskal-Wallis test with Dunn’s multiple comparisons test was applied for comparisons between infection routes (*p < 0.05, **p < 0.01, ***p < 0.001). Correlation analysis of leukocyte population fractions with sepsis score of liver **(C)** and spleen **(E)** is presented as a linear regression graph with a 0.95 confidence interval. Spearman correlation coefficient (r) is indicated. Dots represent individual animals.


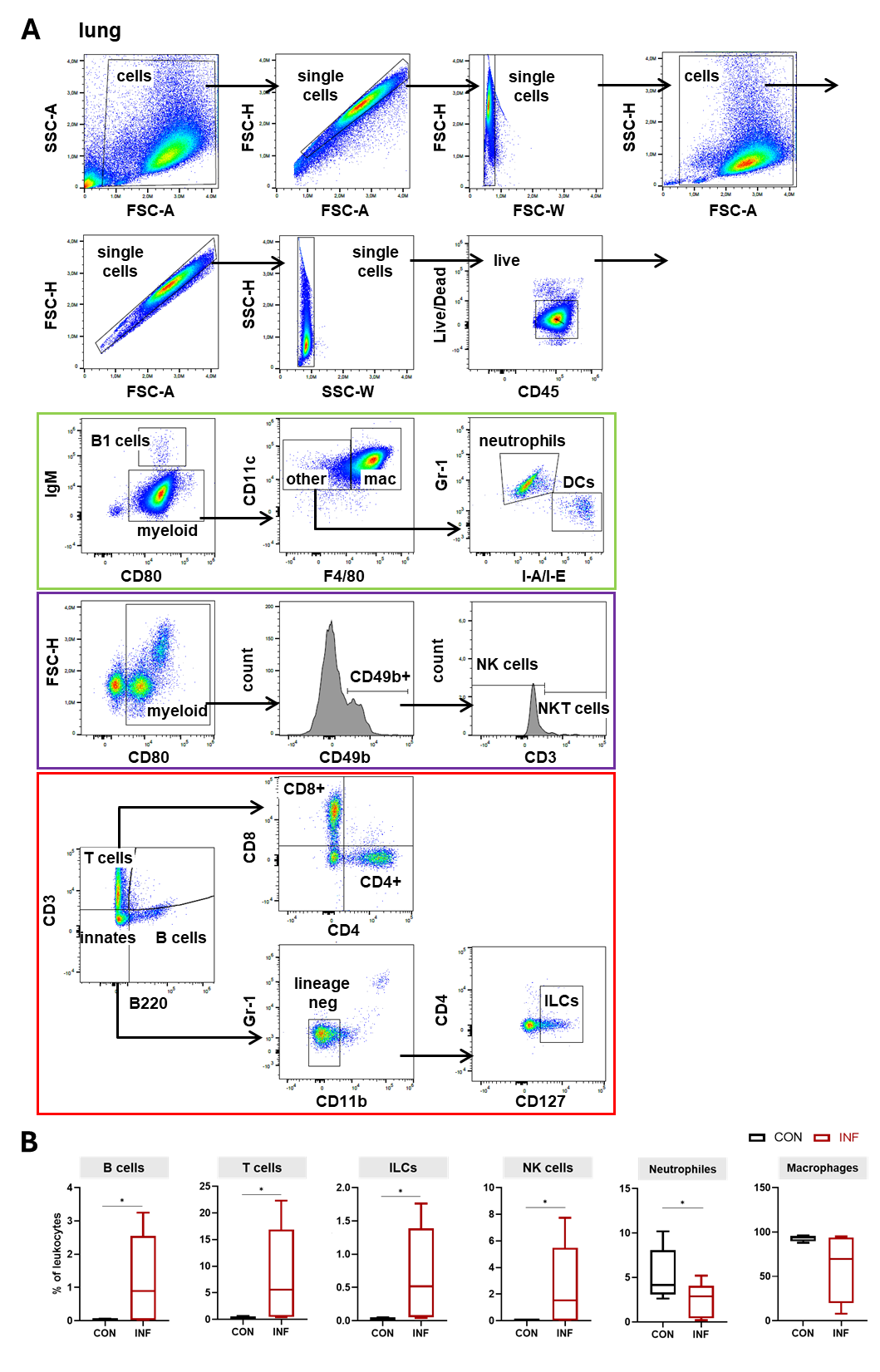


**Supplementary Figure 3. Immune cell landscape in the lung after GAS infection. (A)** Gating strategy for flow cytometry of bronchoalveolar fluid. **(B)** Proportions of leukocyte populations in the lung of intranasally infected mice. Box blots show median values and interquartile range. Mann-Whitney test was used for comparisons between control and infected animals (*p < 0.05).


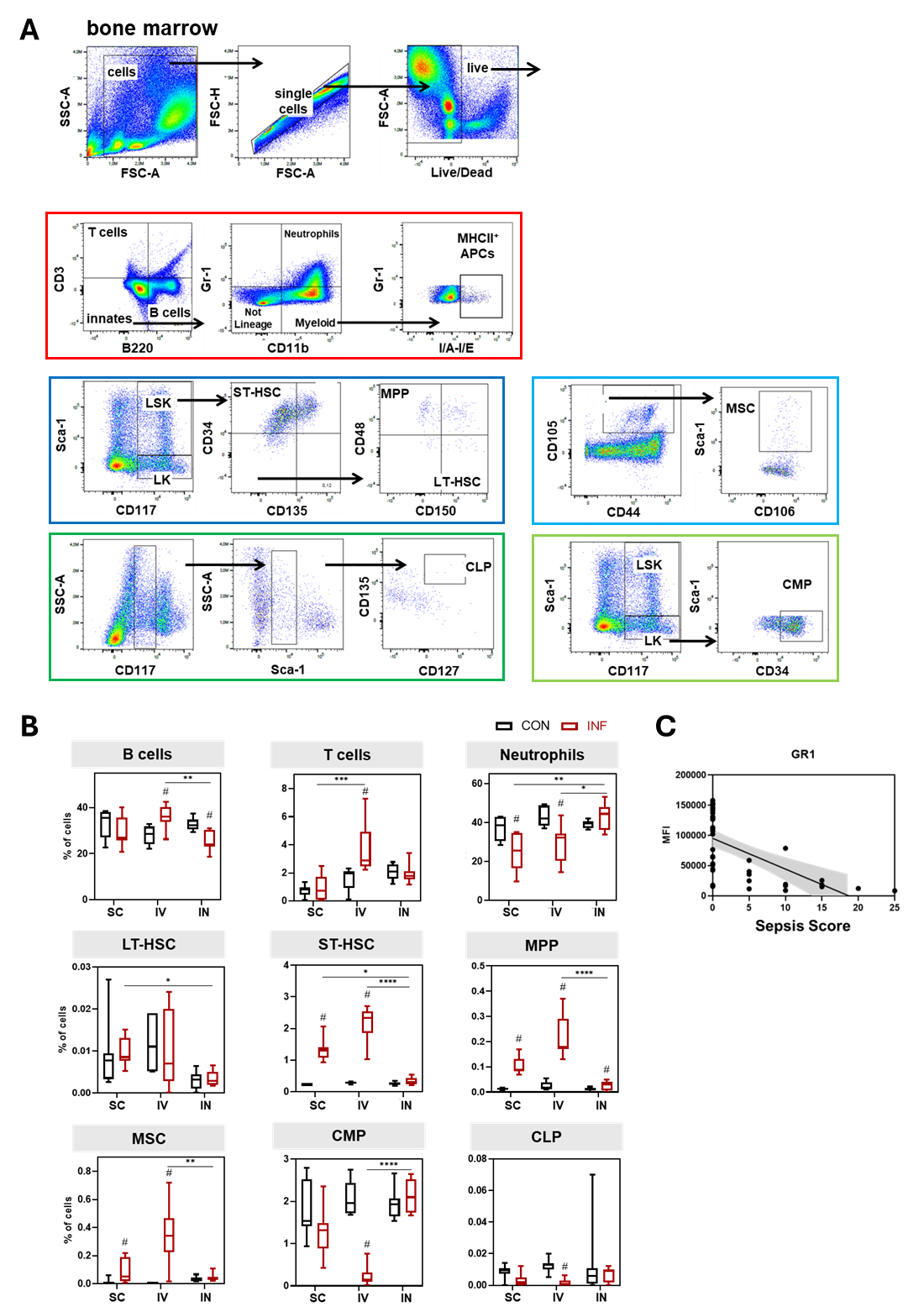


**Supplementary Figure 4. Cell landscape in the bone marrow after GAS infection. (A)** Gating strategy for flow cytometry of the bone marrow compartment. **(B)** Fractions of bone marrow leukocytes and their progenitor cells. Data of control and infected animals are presented for each infection route. Box plots show median values and interquartile range. Mann-Whitney test was used for comparisons between control and infected animals (#p < 0.05). Kruskal-Wallis test with Dunn’s multiple comparisons test was applied for comparisons between infection routes (*p < 0.05, **p < 0.01, ***p < 0.001, ****p < 0.0001). **(C)** Correlation of Gr-1 MFI in neutrophils and sepsis score, presented as a linear regression graph with a 0.95 confidence interval. Dots represent individual values.


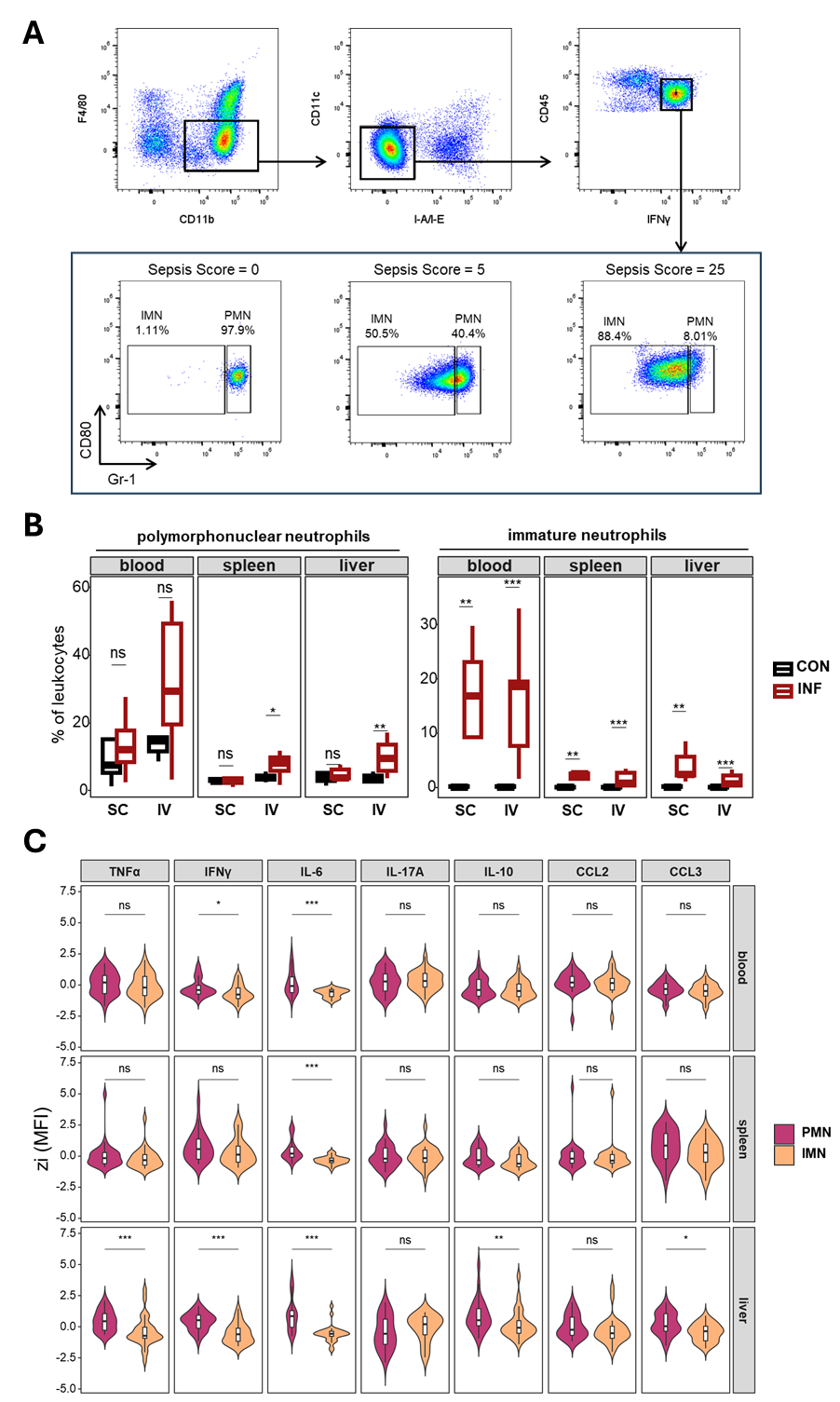


**Supplementary Figure 5. Analysis of immature neutrophils following GAS infection. (A)** Flow cytometry gating strategy for distinguishing immature neutrophils (IMN) from polymorphonuclear neutrophils (PMN). **(B)** Proportions of neutrophil subpopulations in blood, spleen, and liver. Box plots display median values with interquartile ranges. **(C)** Cytokine expression profiles of neutrophil subpopulations in peripheral blood and organs. Violin plots represent the z-values of cytokine expressions. Statistical comparisons between two groups were performed using the Mann-Whitney-U test (*p < 0.05, **p < 0.01, ***p < 0.001).


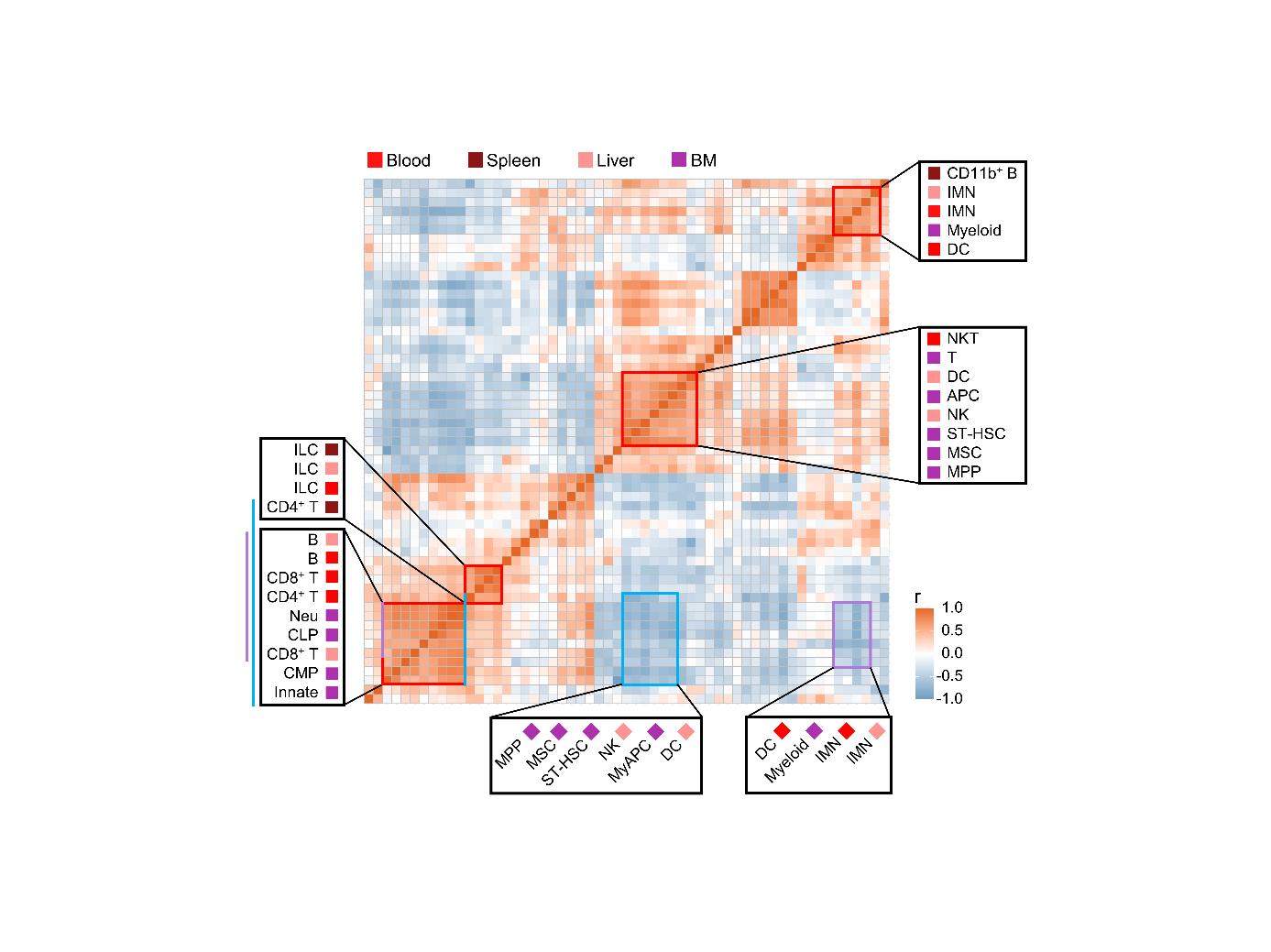


**Supplementary Figure 6: Correlation analysis of the bone marrow compartment and the peripheral immune landscape.** The correlation matrix shows the relationship between frequencies of cells from different organs as shown by differently colored rectangles. Red boxes depict clusters of cell populations that correlate positively with each other. The blue and purple box show clusters of cell populations that correlate negatively with cells that are labeled by the blue and purple bar, respectively. r: Pearson correlation coefficient.


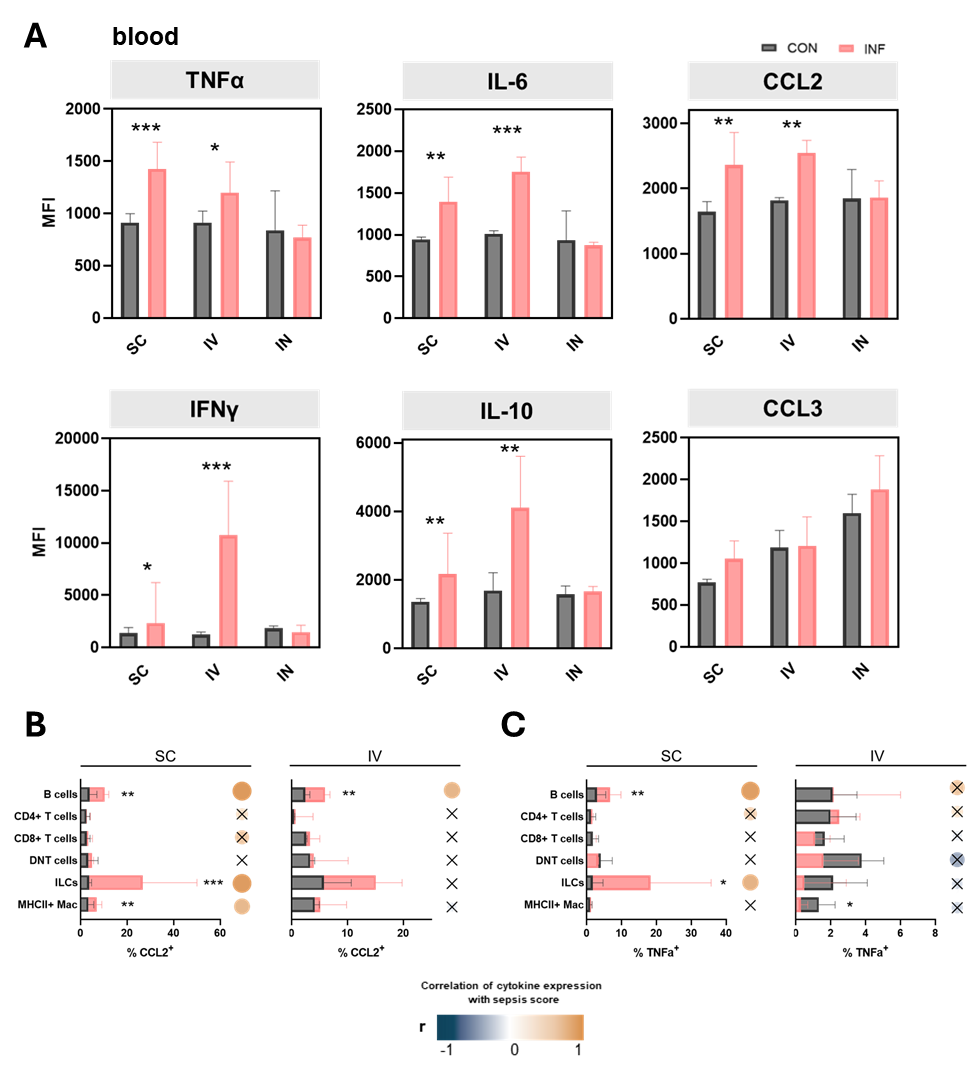


**Supplementary Figure 7. MFI of cytokines in the blood after GAS infection.** Data of control and infected animals are presented for each infection route. Bar plots show median values and interquartile range. Mann-Whitney test was used for comparisons between control and infected animals (*p < 0.05, **p < 0.01, ***p < 0.001).


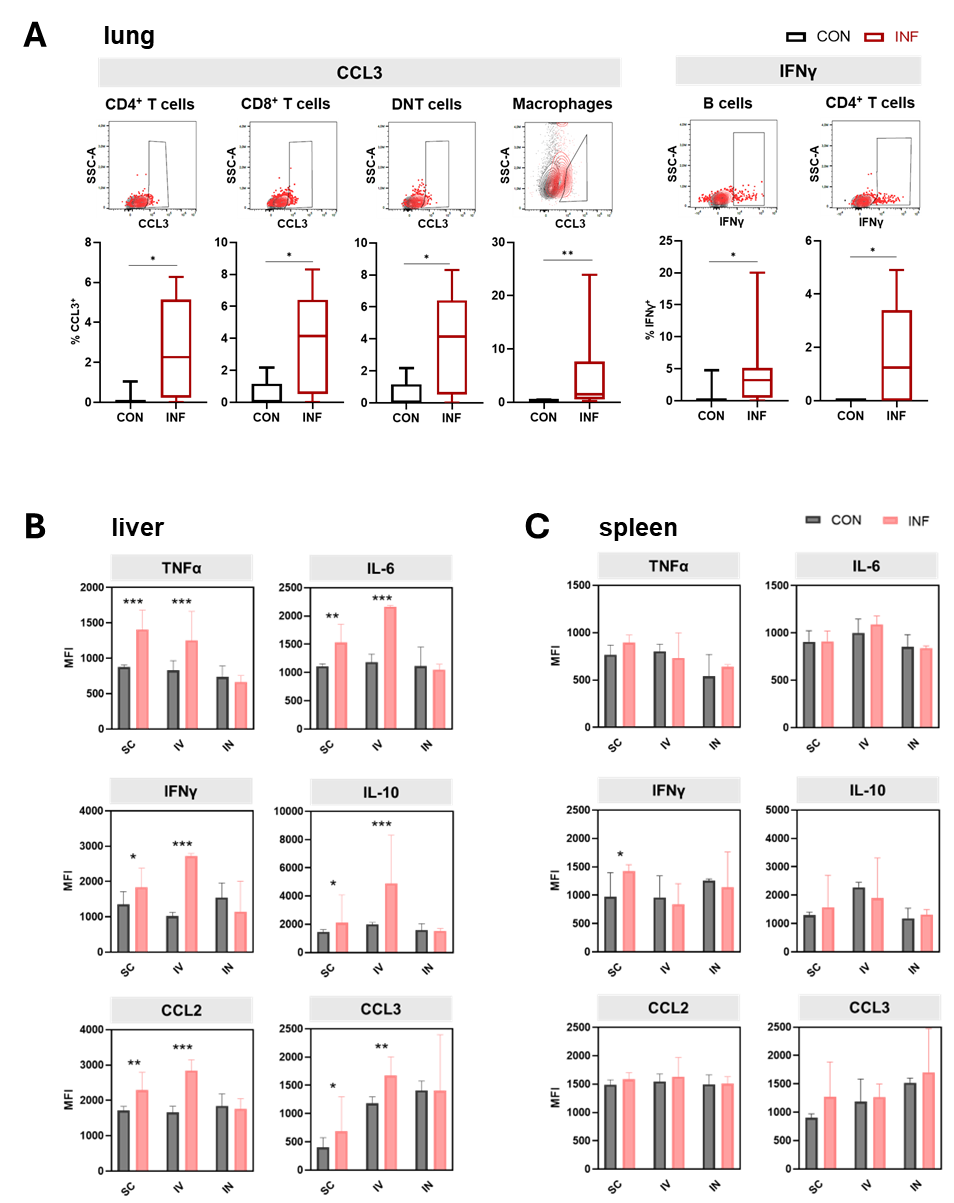


**Supplementary Figure 8. Cytokine analysis in peripheral organs.** MFI of cytokines in liver **(A)** and spleen **(B)**. Data of control and infected animals are presented for each infection route. Bar plots show median values and interquartile range. **(C)** Cytokine expression in the lung after intranasal infection. Gating plots are shown with data of control (black dots) and infected animals (red dots). Box plots show median values and interquartile range. Mann-Whitney test was used for comparisons between control and infected animals (*p < 0.05, **p < 0.01, ***p < 0.001).

**Supplementary Table 1.** **Correlation analysis of cytokine expression by leukocyte populations in the blood with sepsis score in subcutaneous (SC) and intravenous (IV) infection.** Spearman correlation coefficients are listed and values with p < 0.05 are marked in bold.

|  | CCL2 | CCL3 | IFNγ | IL-6 | IL-10 | IL-17A | TNFα |
| --- | --- | --- | --- | --- | --- | --- | --- |
| **SC** |  |  |  |  |  |  |  |
| B cells | **0.7827** | 0.2317 | **0.8061** | **0.8295** | **0.7399** | 0.0000 | **0.7496** |
| B1 cells | **-0.6490** | -0.1505 | -0.1268 | -0.4356 | **-0.5802** | **-0.6507** | -0.4843 |
| NK cells | 0.3427 | 0.0234 | 0.0754 | 0.2819 | 0.0658 | -0.3079 | 0.3076 |
| NKT cells | 0.3635 | -0.0913 | 0.3090 | 0.1413 | 0.1132 | 0.1172 | 0.4195 |
| ILC | **0.7652** | -0.2584 | -0.3456 | 0.1284 | -0.4547 | -0.3456 | **0.5861** |
| DC | 0.4740 | -0.0136 | 0.2439 | 0.0897 | 0.4709 | 0.2024 | 0.2083 |
| MHCII^+^ Mac | **0.5471** | 0.3369 | 0.5009 | **0.5340** | **0.5549** | 0.0253 | 0.1111 |
| Monocytes | 0.1636 | 0.0935 | -0.3563 | **0.5978** | -0.1083 | -0.0704 | 0.1686 |
| Neutrophils | 0.1903 | **-0.7370** | **-0.8066** | 0.3001 | 0.1720 | -0.3367 | -0.2269 |
| CD4^+^ T cells | 0.2940 | 0.0107 | 0.2528 | **0.6211** | 0.1315 | -0.1559 | 0.3719 |
| CD8^+^ T cells | 0.4264 | 0.0740 | 0.4209 | **0.8003** | 0.2181 | -0.2007 | 0.1597 |
| DNT cells | 0.1188 | -0.0254 | 0.1691 | 0.4288 | 0.1325 | 0.2024 | -0.0175 |
|  |  |  |  |  |  |  |  |
| **IV** |  |  |  |  |  |  |  |
| B cells | **0.5674** | 0.3565 | **0.8160** | **0.8519** | **0.7126** | 0.0000 | 0.4497 |
| B1 cells | **-0.6344** | -0.2551 | 0.4795 | -0.0523 | -0.1701 | **-0.6644** | **-0.5875** |
| NK cells | -0.1652 | -0.4438 | **0.7576** | **0.5531** | 0.3265 | 0.1563 | -0.1449 |
| NKT cells | 0.0016 | -0.0556 | **0.8830** | 0.3068 | 0.4726 | 0.0180 | -0.2698 |
| ILC | 0.0065 | 0.0647 | **0.6829** | 0.3736 | 0.0270 | -0.3184 | -0.2083 |
| DC | 0.0507 | 0.4251 | 0.2227 | 0.2862 | -0.0237 | 0.4701 | -0.0630 |
| MHCII^+^ Mac | -0.1472 | **0.5887** | 0.4280 | **0.5511** | 0.3238 | 0.4516 | -0.2365 |
| Monocytes | -0.3041 | **0.5870** | **0.6709** | **0.6099** | 0.1194 | 0.4811 | 0.0476 |
| Neutrophils | -0.2028 | -0.4546 | **-0.5236** | 0.0982 | -0.2911 | 0.2258 | -0.0638 |
| CD4^+^ T cells | 0.0935 | -0.2158 | **0.5928** | **0.7306** | 0.4219 | **0.6619** | 0.2551 |
| CD8^+^ T cells | -0.0196 | 0.2453 | 0.4430 | **0.6655** | 0.2686 | **0.6001** | -0.1432 |
| DNT cells | -0.0932 | -0.0409 | **0.6043** | **0.5302** | 0.1211 | 0.3068 | -0.4377 |

**Supplementary Table 2.** **Correlation analysis of cytokine expression by leukocyte populations in the liver with sepsis score in subcutaneous and intravenous infection.** Spearman correlation coefficients are listed and values with p < 0.05 are marked in bold.

|  | CCL2 | CCL3 | IFNγ | IL-6 | IL-10 | IL-17A | TNFα |
| --- | --- | --- | --- | --- | --- | --- | --- |
| **SC** |  |  |  |  |  |  |  |
| B cells | 0.4420 | 0.4828 | 0.1609 | 0.0526 | **0.7730** | 0.0000 | **0.5802** |
| B1 cells | **0.6976** | 0.2816 | **0.6820** | 0.5075 | **0.8322** | **0.8064** | **0.6802** |
| NK cells | 0.3869 | 0.1887 | 0.3976 | 0.3274 | 0.4868 | **0.5869** | **0.7093** |
| NKT cells | 0.4794 | **0.6900** | **0.5344** | -0.0078 | **0.7328** | **0.6365** | **0.5796** |
| ILC | 0.1438 | **0.6205** | 0.4279 | 0.1832 | **0.5726** | -0.1866 | 0.3674 |
| DC | **0.5493** | 0.2142 | -0.0586 | 0.1538 | **0.5627** | **0.5830** | **0.5730** |
| MHCII^+^ Mac | 0.4128 | -0.0175 | -0.4576 | 0.2329 | **0.5822** | 0.3235 | **0.5394** |
| Monocytes | **0.5802** | 0.1090 | -0.3738 | 0.4809 | **0.6562** | 0.4873 | **0.5622** |
| Neutrophils | **0.6343** | **0.5934** | **-0.7150** | 0.1720 | -0.1647 | **0.6082** | 0.4282 |
| CD4^+^ T cells | 0.3407 | **0.7676** | **0.7269** | 0.3255 | **0.7406** | 0.5120 | 0.4732 |
| CD8^+^ T cells | 0.3761 | 0.2570 | **0.5246** | 0.2183 | **0.7641** | **0.7257** | 0.4229 |
| DNT cells | 0.3508 | **0.6365** | 0.4553 | 0.3771 | 0.3941 | **0.5628** | 0.4084 |
|  |  |  |  |  |  |  |  |
| **IV** |  |  |  |  |  |  |  |
| B cells | 0.4955 | 0.3728 | **0.8460** | 0.4104 | **0.8057** | 0.0000 | 0.4336 |
| B1 cells | 0.3674 | 0.0213 | 0.3581 | **0.5756** | **0.7129** | **0.5380** | 0.4317 |
| NK cells | -0.3085 | 0.4153 | **0.7555** | -0.0932 | **0.6387** | **0.7527** | -0.0426 |
| NKT cells | -0.4301 | **0.7113** | 0.1880 | **-0.6611** | 0.3597 | 0.0033 | 0.1244 |
| ILC | 0.2365 | 0.4297 | **0.7459** | -0.3184 | 0.2351 | 0.0000 | 0.2664 |
| DC | -0.4121 | **0.5134** | 0.4005 | 0.4039 | **0.5674** | **0.7669** | 0.1357 |
| MHCII^+^ Mac | **-0.7178** | **0.6786** | 0.4181 | **-0.7522** | 0.4168 | **0.7495** | **0.6394** |
| Monocytes | 0.2207 | **0.7309** | 0.5968 | 0.0614 | **0.6603** | **0.7672** | 0.4529 |
| Neutrophils | 0.4385 | 0.2355 | **-0.6737** | 0.1112 | 0.0180 | **0.5940** | **0.5122** |
| CD4^+^ T cells | 0.1880 | **0.8634** | **0.7675** | 0.2633 | 0.2665 | 0.4389 | 0.4693 |
| CD8^+^ T cells | 0.2594 | **0.8241** | **0.7828** | 0.2813 | 0.4858 | 0.4205 | 0.2260 |
| DNT cells | 0.0245 | **0.8331** | **0.7216** | 0.3205 | 0.2649 | **0.5838** | -0.1268 |
